# Prevalence and Patterns of Antimicrobial Resistance among *Escherichia coli* and *Staphylococcus* spp. in a Veterinary University Hospital

**DOI:** 10.3390/vetsci8120308

**Published:** 2021-12-06

**Authors:** Giorgia Cocca, Silvia Piva, Sara Del Magno, Raffaele Scarpellini, Federica Giacometti, Andrea Serraino, Massimo Giunti

**Affiliations:** Department of Veterinary Medical Sciences, Alma Mater Studiorum-University of Bologna, Ozzano Emilia, 40064 Bologna, Italy; giorgia.cocca@studio.unibo.it (G.C.); silvia.piva@unibo.it (S.P.); sara.delmagno@unibo.it (S.D.M.); raffaele.scarpellin2@unibo.it (R.S.); federica.giacometti3@unibo.it (F.G.); andrea.serraino@unibo.it (A.S.)

**Keywords:** intensive care unit, dogs, cats, carbapenems, multi drug resistance

## Abstract

The occurrence of antimicrobial resistance in commensal strains of *Escherichia coli* and *Staphylococcus* spp. was investigated in 320 samples collected from patients and the environment of a veterinary university hospital—specifically, the consultation area (CA) and intensive care unit (ICU). *E. coli* was isolated in 70/160 samples (44%), while *Staphylococcus* spp. were isolated in 110/160 (69%) samples. The occurrence of multidrug-resistant (MDR) isolates from CA and ICU admission were similar for *E. coli* (1/12 (8%) versus 4/27 (15%), respectively) and *Staphylococcus* spp. (10/19 (53%) versus 26/50 (52%), respectively). MDR *E. coli* isolates increased significantly at hospital discharge (18/31; 58%; *p* = 0.008). Antimicrobial treatment administered during hospitalization was a risk factor for carriage of MDR *E. coli* (OR, 23.9; 95% CI: 1.18–484.19; *p* = 0.04) and MDR *Staphylococcus* spp. (OR, 19.5; 95% CI 1.30–292.76; *p* = 0.02), respectively. The odds ratio for MDR *E. coli* was 41.4 (95% CI 2.13–806.03; *p* = 0.01), if the administration of fluoroquinolones was evaluated. The *mecA* gene was detected in 19/24 (79%) coagulase-positive *Staphylococcus* spp. isolates resistant to oxacillin. High rates of MDR *Staphylococcus* spp. were reported. Hospitalization in the ICU and antimicrobial treatment were risk factors for colonization by MDR commensal bacteria.

## 1. Introduction

Antimicrobial resistance (AMR) is a natural phenomenon accelerated by the abuse and misuse of antimicrobial drugs in human and veterinary medicine and is considered by the World Health Organization as one of the most important current public health threats [1,2]. Among companion animals, AMR represents an emerging problem that involves both commensal and pathogenic bacteria [3]. In recent years, several multidrug-resistant (MDR) bacteria have emerged in dog and cat populations, constituting a serious threat due to possible therapeutic failure, longer hospitalization periods, increased treatment costs, and morbidity [4,5,6]. Antimicrobial treatment, length of hospitalization, and particularly intensive care unit (ICU) stays are associated with increased rates of antimicrobial resistance genes in commensal bacteria, including *Escherichia coli* and *Staphylococcus* spp. [7,8,9,10,11]. *E. coli* is a representative indicator of antimicrobial resistance in gram-negative bacteria [12]. Staphylococci are commensal bacteria living on the skin and in the mucous membranes of different hosts and can often act as opportunistic pathogens. The prevalence of antimicrobial resistance in *Staphylococcus* spp., particularly methicillin resistance, has risen in recent decades in both human and veterinary patients, and is a focus of the antibiotic resistance surveillance program in food-producing animals [13,14]. The spread of AMR within veterinary hospitals constitutes a serious public health threat due to potential interspecies transmission, including humans [4,15,16,17].

The aim of the present study was to evaluate the prevalence and pattern of antimicrobial resistance of commensal strains of *E. coli* and *Staphylococcus* spp. isolated from dogs and cats and from the environment in different areas of a veterinary university hospital (VUH). The development of AMR during hospitalization was also evaluated in order to investigate the potential influence of antibacterial treatment. Our hypothesis was that rates of MDR bacterial isolates increase during hospitalization in patients treated with antibiotics and are higher compared to non-treated dogs and cats from the CA.

## 2. Materials and Methods

### 2.1. Population and Enrolment Criteria

This study was carried out from January 2018 to January 2019 using a sample population of dogs and cats coming from two different areas of a VUH, namely, the CA and the ICU, representing two different groups. Patients from the CA included dogs and cats undergoing routine visits who had not received antimicrobial treatment for at least one month prior to sampling and were classified as group one. The second group included dogs and cats hospitalized in the ICU for at least 24 h. Data collected for all hospitalized patients included signalment, diagnosis, length of hospitalization, and antimicrobial treatment received in the last 30 days and/or administered during hospitalization.

### 2.2. Sample Collection

For patients from the CA (group one), rectal and oropharyngeal swabs were performed on each animal for the isolation of *E. coli* and *Staphylococcus* spp., respectively. Rectal swabs were inserted about 2 cm into the rectum and gently rotated to facilitate fecal material adhesion. For oropharyngeal swabs, after opening the mouth of the animal, a vigorous swab of the back of the oropharynx was performed. At the end of the patient examination, environmental surface sampling (e.g., table surface, stethoscope, computer keyboard) was performed using a sterile sponge for the isolation of both *E. coli* and *Staphylococcus* spp. A similar protocol was applied for patients from the ICU (group two), both at the time of admission and at hospital discharge, including environmental sampling of the surface of the ICU cage. After sampling, oropharyngeal and rectal swabs were immediately placed in the AMIES transport medium (AMIES transport medium, APTACA S.R.L, Asti, Italy), while environmental sponges were placed in a sterile stomacher bag containing 100 mL of sterile 0.9% NaCl solution. All samples were stored at 4 °C ± 2 for a maximum of 24 h.

### 2.3. Sample and Population Composition

In this study, a total of 320 samples were collected, 160 from patients and 160 from the environment. Samples were obtained from a total of 48 patients: 32 dogs (13 females and 19 males) with a median age of 7.5 years (1–16) and a median weight of 13.5 kg (2–36), and 16 cats (5 females and 11 males) with a median age of 7 years (2–14) and a median weight of 5 kg (2–7). Group one included 16 patients coming from the CA, including 14 dogs (8 referred for routine prophylaxis and 6 presented for first opinion in internal medicine) and two cats, referred for kidney and gastrointestinal diseases, respectively. For group two, 32 patients were hospitalized in the ICU, including 18 dogs with the following diseases: nephropathy (4/18), infectious disease (4/18), gastroenteropathy (3/18), endocrinopathies (2/18), trauma (2/18), anemia (2/18), and neoplasia (1/18). Furthermore, 14 cats were hospitalized for neoplasia (3/14), nephropathy (2/14), infectious disease (2/14), endocrinopathies (2/14), trauma (2/14), anemia (1/14), cardiomyopathy (1/14), and gastroenteropathy (1/14). Most patients from the ICU (27/32, 84%) received antimicrobial therapy during hospitalization and 6/32 (19%) were treated with antimicrobials in the last 30 days prior to hospitalization. The majority of patients included in the study received enhanced penicillins (13/27, 48%), followed by fluoroquinolones (10/27, 37%) and cephalosporines (6/27, 22%). The median hospitalization period was 3.5 days (1–29) for dogs and 4 days (1–8) for cats.

### 2.4. Bacteria Isolation and Identification

Rectal and oropharyngeal swabs were plated onto McConkey Agar (MAC, Thermo Fisher Scientific, Milan, Italy) and Mannitol Salt Agar (MSA, Thermo Fisher Scientific, Milan, Italy), respectively. Plates were incubated aerobically at 37 °C ± 1 for 24–48 h. After growth, the presumptive *E. coli* and *Staphylococcus* spp. colonies were subcultivated on Tryptic Soy Agar (TSA, Thermo Fisher Scientific, Milan, Italy) and frozen at −80 °C for further examination. DNA extraction was performed using the REDExtract-N-Amp Tissue polymerase chain reaction (PCR) kit (Sigma-Aldrich, Milan, Italy). Presumptive *E. coli* were identified using the PCR procedure described by Clermont and colleagues [18]; in case of doubtful results, as well as for all staphylococci identifications, the isolates were subjected to PCR for the amplification of a portion of the 16S rRNA gene using generic primers p27-f (5′-AGAGTTTGATCCTGGCTCAG-3′) and 1492-r (5′-TACGGCTACCTTGTTTTACGACT-3′). The 833 bp PCR products were sequenced by the Eurofins Scientific laboratory using the Sanger method. Sequences were corrected and analyzed using Bioedit and MEGA sequence editor software, and then compared with those filed in international databases using the Basic Local Alignment Search Tool (BLAST) to obtain bacterial identification [19,20,21].

### 2.5. Antimicrobial Susceptibility Testing

The antimicrobial susceptibility of all the isolates was determined using the Kirby–Bauer disk diffusion method according to the Clinical and Laboratory Standard Institute (CLSI) method [22,23]. To evaluate the epidemiological aspects of antimicrobial resistance patterns, the epidemiological breakpoints proposed by the European Committee on Antimicrobial Susceptibility Testing were used as the first choice, when available. For missing values, antimicrobial breakpoints proposed by CLSI were alternatively selected. Intermediate susceptibility to antimicrobials was allotted to the resistance category. MDR was defined as resistance to three or more antimicrobial categories [24].

### 2.6. mecA PCR

The presence of the *mecA* gene was investigated in all the isolated coagulase-positive oxacillin-resistant staphylococci using PCR according to the modified protocol of Stegger et al. [25]. Each PCR contained 25 μm of the *mecA* primer (mecA-P1-5′-TCCAGATTACAACTTCACCAGG-3′; and mecA-P2-5′-CCACTTCATATCTTGTAACG-3′) (162pb), 1x REDExtract-N-Amp PCR ReadyMix (Sigma-Aldrich, Milan, Italy) and 2 μL of DNA template preparation. DNA of *Staphylococcus aureus* ATCC 48866 and water were used as positive and negative controls, respectively.

### 2.7. Statistical Methods

Descriptive statistical analysis was carried out to describe the occurrence and pattern of antimicrobial resistance. Univariate linear regression analysis was used to evaluate significant variables with respect to MDR carriage at admission and discharge. The odds ratios (ORs) and the 95% confidence intervals (CIs) for the categorical variables significantly associated with MDR were calculated. The rates of resistance between admission and discharge were tested with the McMenar Test. Significance was set at *p* < 0.05. Statistical analysis was performed using statistical software (MedCalc^®^ Statistical Software version 19.6, MedCalc Software Ltd., Ostend, Belgium, 2020).

## 3. Results

### 3.1. Detection of E. coli and Staphylococcus spp.

The findings concerning data related to the isolation of *E. coli* and *Staphylococcus* spp. in all samples are reported in Table 1. Overall, *E. coli* was isolated in 70/160 samples (44%), while *Staphylococcus* spp. were isolated in 110/160 (69%) samples, with *Staphylococcus pseudintermedius* being the most frequently isolated species (35/110, 32%; Table 2).

### 3.2. Occurrence and Pattern of Antimicrobial Resistance

Nine out of 12 (75%) *E. coli* isolates in animals from the CA were susceptible to all drugs tested and only 1/12 (8%) was classified as MDR. Overall, higher rates of resistance were observed in *Staphylococcus* spp. isolates from the CA with 16/19 (84%) being resistant to at least one drug and 10/19 (53%) classified as MDR, equally distributed between patients and environment. Data related to the incidence rates of antimicrobial resistance in *E. coli* isolates at ICU admission and discharge are reported in Table 3.

Apart from chloramphenicol, rates of resistance increased significantly for all antimicrobial molecules tested during hospitalization. Interestingly, *E. coli* isolates resistant to imipenem upon admission showed a rate of 1:27 (4%) which increased to 1:3 (10/31; 32%) at patient discharge (*p* = 0.01), despite this drug not being used in the VUH during the study period. Overall, 17/27 (63%) of *E. coli* isolates were susceptible to all drugs tested upon admission to ICU, while 10/27 (37%) were resistant to at least one drug. At hospital discharge, the incidence rates of *E. coli* isolates susceptible to all drugs tested (9/31; 29%) and resistant to at least one drug (22/31, 71%), respectively, decreased and increased compared to those reported upon admission, but these changes were not significant. Concerning MDR *E. coli* isolates over the total number of isolates, an incidence rate of 1:7 was reported in patients at ICU admission (4/27; 15%)—a figure not significantly different from that reported for animals in the CA. However, MDR *E. coli* isolates increased significantly to a rate of 1:2 in patients at hospital discharge (18/31; 58%) (*p* = 0.008). Moreover, two out of the only three environmental *E. coli* isolates from ICU cages at patient discharge (see Table 1) were MDR. Univariate analysis identified the following variables as risk factors for MDR carriage in *E. coli*: antimicrobial treatment administered during hospitalization in ICU (OR, 23.9; 95% CI 1.18–484.19; *p* = 0.04) and, specifically, the administration of fluoroquinolones (OR, 41.4; 95% CI 2.13–806.03; *p* = 0.01).

Data related to the incidence rates of antimicrobial resistance in *Staphylococcus* spp. isolates in patients at ICU admission and discharge are reported in Table 4.

Regarding the *Staphylococcus* spp. isolates upon admission to ICU, 10/50 (20%) were susceptible to all drugs tested, 40/50 (80%) showed resistance to at least one drug, while 26/50 (52%) were MDR. Previous antimicrobial treatment of patients was associated with the presence of MDR in 7/9 (78%) *Staphylococcus* spp. isolates, but this association was not statistically significant (*p* = 0.08). For *Staphylococcus* spp. isolates, univariate analysis identified the administration of antimicrobial treatment during hospitalization in ICU as a risk factor for MDR carriage (OR, 19.5; 95% CI 1.30–292.76; *p* = 0.02). *Staphylococcus* spp. isolates at patient discharge showed similar rates of resistance to those reported at ICU admission with 8/41 (20%) susceptible to all drugs tested, 33/41 (80%) resistant to at least one drug, and 27/41 (66%) being MDR. According to these findings, the rates of resistance for *Staphylococcus* spp. isolates of the single antimicrobial molecule tested presented little variation during hospitalization and were not statistically significant compared to ICU admission (Table 4). The presence of the *mecA* gene was detected in 19/24 (79%) coagulase-positive *Staphylococcus* spp. (CoPS) isolates resistant to oxacillin, namely 15 *S. pseudintermedius* (all isolates from ICU) and 4 *S. aureus* (three from the ICU and one from the CA) (Table 5).

## 4. Discussion

This prospective observational study describes the incidence rate and pattern of antimicrobial resistance among commensal *E. coli* and *Staphylococcus* spp. isolated from dogs and cats and from the environment in two areas (the consultation area and the intensive care unit) of a VUH—areas for which a difference in the risk of colonization/infection by AMR bacteria (low and high, respectively) is expected, based on the impact of antimicrobial selection pressure and severity of illness. 

A low prevalence of *E. coli* isolates (3/32, 9%) was reported from ICU cages at patient discharge, suggesting that disinfection procedures were carried out correctly in the investigated VUH. However, the sampling site was limited to ICU cages, and we could not exclude a higher rate of *E. coli* isolation from other sites (e.g., floor surfaces, equipment such as thermometers, and areas of high human hand contact, such as telephones and doorknobs), as previously reported [26]. In agreement with previous data from the veterinary literature, our findings confirmed moderate to low rates of resistance characterizing *E. coli* isolated from patients without a recent history of antimicrobial therapy, with a low percentage of them being MDR [7,27]. Moreover, the most frequent patterns of resistance in *E. coli* isolates were observed for aminoglycosides (17%), β-lactams (8%), and tetracycline (8%), as previously reported [7,27,28,29]. Not surprisingly, the frequency of observed resistance and MDR were higher in *E. coli* isolates from hospitalized patients than those from consultations; the most common patterns of resistance in the ICU were recorded for penicillins, fluoroquinolones, tetracycline, and trimethoprim-sulfamethoxazole (see Table 3). These patterns of antimicrobial resistance partly resemble data from similar studies in hospitalized cats and dogs, documenting increased resistances for ampicillin, tetracycline, trimethoprim–sulfamethoxazole, and quinolones [30,31]. Furthermore, our results showed that resistance to at least one drug and MDR increased significantly during ICU stays—data in line with previous studies showing that hospitalization, particularly in the ICU, negatively affects patterns of resistance among *E. coli* isolates both in human and veterinary hospital settings [8,9,10]. Higher rates of AMR, including extended-spectrum-β-lactamase (ESBL) phenotype strains, are reported in the ICU compared to non-ICU units, and are related to several risk factors, such as increased use of broad-spectrum antimicrobials, invasive procedures, and a greater possibility of bacteria transmission between patients [32]. Interestingly, we found a not negligible and worryingly increased rate of imipenem-resistant *E. coli* isolates in our patients during hospitalization. The presence of hospital-acquired carbapenem-resistant *E. coli* poses a serious issue concerning the possibility of dissemination into the environment and transmission to other animals and humans [4,33]. The potential cause behind the isolation of carbapenem-resistant *E. coli* in our study was not investigated but could be related to the presence of carbapenemase-producing bacteria, since hospitalized pets can represent a possible source of carbapenem-resistant gram-negative bacteria [34,35]. Furthermore, the upregulation of efflux pumps mediated by fluoroquinolones, capable also of extruding carbapenems from the bacterial cell, is another reasonable hypothesis [36]. The latter can be corroborated by the facts that fluoroquinolones were among the most used drugs in our VUH and that all isolates resistant to carbapenems were also resistant to fluoroquinolones. 

Finally, we observed a significant association between antimicrobial therapy received during hospitalization and the detection of MDR *E. coli*, as well as between fluoroquinolone administration during ICU stays and the isolation of fluoroquinolone-resistant (FQR) *E. coli*. These findings are consistent with previous studies in which the use of fluoroquinolones increased the risk of detecting FQR and MDR *E. coli* [8,10,37,38,39].

Regarding *Staphylococcus* spp. isolates, *S. pseudintermedius* was the most frequently observed. This is consistent with a previous report, as this species normally colonizes the skin and mucosal sites of companion animals [40]. Regardless of risk category, *Staphylococcus* spp. isolates showed high rates of AMR with resistance to at least one drug and MDR was found in 80% and 50% of isolates, respectively. These findings were expected, since moderate to high resistance rates often characterize *Staphylococcus* spp. isolates from previously untreated pets [7,41,42]. A study on the evolution of antimicrobial resistance in *Staphylococcus* spp. isolated from companion animals showed a significant increase in resistance to the majority of antimicrobials and in the number of *mecA*-positive isolates over a 16-year period [43]. A similar result was obtained by Detwiler and colleagues, who reported an increase in *Staphylococcus* spp. MDR strains over a six-year period, in addition to the recognition of extensively drug-resistant (XDR) *S. pseudintermedius* [44]. The main patterns of resistance found in our study (see Table 4) were against lincosamides, aminoglycosides, β-lactams, tetracycline, and trimethoprim–sulfonamide, in line with other studies [5,44].

With respect to *E. coli*, environmental isolation of staphylococci was higher, but this result is not surprising since they constitute part of the normal microbial flora of both humans and animals, are widely spread in a veterinary hospital environment, and possess a long period of survival time in hospital settings and fabrics [45,46,47,48].

Methicillin-resistant *S. pseudintermedius* (MRSP) and methicillin-resistant *Staphylococcus aureus* (MRSA) were identified in our patients and on environmental surfaces both in the ICU and the CA. Methicillin resistance is conferred by the *mecA* gene which codes for a new “penicillin-binding protein” that confers resistance to all β-lactam antimicrobials [49]. In addition, methicillin resistance is often associated with resistance to lincosamides, fluoroquinolones, macrolides, tetracyclines, and trimethoprim–sulfonamides [50]. Studies on the prevalence of MRSP and MRSA colonization in small animals showed rates ranging from 0% to 4.6% [28,49,51,52,53,54,55,56]. Higher rates of MRSP and MRSA carriage are reported in hospitalized patients and in the hospital environment [57]. Risk factors associated with MRSP infections include hospitalization, topical ear medications, glucocorticoid therapy, antimicrobial therapy, and the presence of skin lesions [58,59]. Higher rates of methicillin-resistant isolates from hospitalized compared to non-hospitalized patients were also observed in the present study; in fact, only 1/19 (5%) MRSA were derived from the CA while 3/19 (16%) MRSA and 15/19 (79%) MRSP came from the ICU (*p* = 0.0001). Methicillin resistance detected in the VUH could be linked to the selection pressure exerted by the use of β-lactams and fluoroquinolones, since these drugs are the most widely used in the VUH and the most prescribed by veterinarians in the region where the study was carried out [60].

This study had some limitations, the main one being the small sample size of the investigated population. Furthermore, besides patient history of antibacterial treatment, other potential sources or risk factors associated with the carriage of MDR bacteria were not explored in the animals included in the study. Finally, a defined daily dose to monitor antibiotic consumption in our VUH was not available in the study period.

## 5. Conclusions

The present study documented a significant occurrence of MDR *E. coli*, MRSA, and MRSP isolates from dogs and cats during hospitalization in the ICU compared to community pets from the CA of the hospital. Antimicrobial treatment during hospitalization was confirmed as a risk factor for the carriage of MDR commensal bacteria. Furthermore, a significant report of carbapenem-resistant *E. coli* isolates in patients at the end of hospitalization constitutes a public health concern. Finally, the institution of infection control programs, including surveillance systems and an antimicrobial stewardship approach in veterinary facilities are highly recommended to prevent the emergence and spread of antimicrobial resistance.

## Figures and Tables

**Table 1:** Descriptive data regarding the isolation of *E. coli* and *Staphylococcus* spp. from the rectal and oropharyngeal swabs performed, respectively, and from the environmental surfaces in the consultation area (CA) and intensive care unit (ICU) of the veterinary hospital.

Hospital Area	No. of Patient Swabs	Isolates *n* (%)	N. of Environment Swabs	Isolates *n* (%)	Total Isolates *n* (%)
*Escherichia coli*					
CA	16	12 (75%)	16	-	12 (37%)
ICU admission	32	27 (84%)	32	-	27 (42%)
ICU discharge	32	28 (87%)	32	3 (9%)	31 (48%)
	*Staphylococcus* spp.				
CA	16	9 (56%)	16	10 (62%)	19 (59%)
ICU admission	32	23 (72%)	32	27 (84%)	50 (78%)
ICU discharge	32	20 (62%)	32	21 (66%)	41 (64%)

**Table 2:** Number of different staphylococcal species isolated from oropharyngeal and environmental samples.

Staphylococcal Species	Coagulase	No. of Isolates	%
*S. pseudintermedius*	Positive	34	30
*S. haemolyticus*	Negative	3	10
*S. aureus*	Positive	7	6
*S. epidermidis*	Negative	7	6
*S. felis*	Negative	6	5
*S. hominis*	Negative	3	3
*S. cohnii*	Negative	2	2
*S. simulans*	Negative	2	2
*S. vitulinus*	Negative	1	1
*S. capitis*	Negative	1	1
*S. succinus*	Negative	1	1
*S. xylosus*	Negative	1	1
*Staphylococcus* spp.	/	32	29
Total		110	69

Legend: the symbol / indicates non-difinable coagulase.

**Table 3:** Incidence rates of antimicrobial resistance in *E. coli* isolates in animals and the environment at ICU admission and discharge.

Antimicrobial Agent	Admission (Total Isolates, *n* = 27)		Discharge (Total Isolates, *n* = 31)		*p*
	R	%	R	%	
AMP	6	22	20	65	0.02
AMC	3	11	16	52	0.007
CAZ	4	15	14	45	0.04
CZ	3	11	14	45	0.02
IMP	1	4	10	32	0.01
GMN	2	7	14	45	0.006
C	1	4	5	16	0.14
ENR	4	15	17	55	0.01
NA	4	15	18	58	0.008
TE	6	22	18	58	0.03
SXT	4	15	16	52	0.02
MDR	4	15	18	58	0.009

**Table 4:** Incidence rates of antimicrobial resistance in *Staphylococcus* spp. isolates in animals and the environment at ICU admission and discharge.

Antimicrobial Agent	Admission (Total Isolates *n* = 50)		Discharge Total Isolates *n* = 41)		*p*
	No.	%	No.	%	
AMP	31	62	24	59	0.83
AMC	15	30	12	29	0.95
OX	25	50	16	39	0.44
KF	16	32	11	27	0.65
EFT	18	36	13	32	0.73
IMP	3	6	0	0	0.12
S	22	44	24	59	0.33
GMN	20	40	17	41	0.91
DA	27	54	26	63	0.56
ENR	18	36	17	41	0.68
E	28	56	27	66	0.55
TE	22	44	22	54	0.51
SXT	14	28	14	34	0.60
MDR	26	51	27	66	0.39

**Table 5:** Incidence of *mecA* gene detected in 24 oxacillin-resistant CoPs isolated from CA and ICU.

OX-Resistant CoPS	CA	ICU
**OX-resistant** ***Staphylococcus aureus***	1/24	3/24
**OX-resistant** ***Staphylococcus pseudintermedius***	0/24	15/24

## Data Availability

Data is contained within the article (tables and dot plots).
